# A Case of Atresia Vaginæ

**Published:** 1842-01-01

**Authors:** Ralph C. Marsh

**Affiliations:** Delaware Co., Pa.


### MEDICAL EXAMINER.

NEW SERIES. — Vol. V

No. 1.] PHILADELPHIA, JAN. 1, 1842. [Vol. I.

Surgeon Gen's Office LIBRARY Washington, D.C.

### ORIGINAL COMMUNICATIONS.

*A Case of Atresia Vaginæ.* By Ralph C. Marsh, M. D. of Delaware Co., Pa. *Communicated, with remarks, by* C. D. Meigs, M. D., Professor of Midwifery in the Jefferson Medical College.

To the Editors of the Medical Examiner.

Gentlemen,—I received a letter, dated 30th November, from Dr. Ralph C. Marsh, a gentleman of great experience and judgment, who is now engaged in the practice of medicine at Concordville, Delaware Co., Penn., where he has long been in practice. As the letter contains the relation of a singular case, I take the liberty of handing it to you for publication in your valuable Journal.

What renders the history the more interesting, is the circumstance that a complete atresia vaginæ should take place in a single woman, after menstruating regularly for years. The case that fell under the notice of Dr. Randolph and myself, and which was published in the Philadelphia Practice of Midwifery,—as well as a very similar one detailed in the recent valuable treatise on midwifery, by M. Moreau,—were the results of vaginal inflammation and sloughing, consequent on labour.

In Dr. Marsh's case it does not appear that any injury or violence had been noticed as the cause of the patient's difficulty.

Yours truly,

C. D. Meigs.

The case is stated as follows:

A. H., a young woman, aged 18 years, of large size and strong health, was, in the spring of 1823, attacked with symptoms of a disorder resembling dysmenorrhœa. Dr. S., who was the attending physician of the family, being applied to, treated her, so far as I could learn, as for painful menstruation. The symptoms were unusually severe and continued, as is usual in such cases; three or four days after this, she recovered and was able to pursue her ordinary business of house-work in her father's family.

Upon the return of the menstrual period, she was again seized with similar symptoms, and even more violently than before. The physician being recalled, gave his attendance, and she again recovered and resumed her occupations. By Dr. S.'s advice, it was decided that if she should have another attack I should be called to the case. On the approach of another period the distressing symptoms reappeared, and I saw her. I treated her in the ordinary way, with venesection, sudorifics, and opiates, so that the spasms, &c. were again relieved.

Upon inquiry, I found that she had menstruated at an early period of life, indicating an early development of the reproductive apparatus. This first menstruation had occurred three or four years before, and the health and menstruation were perfect until within a few months before the appearance of these distressing symptoms. I left the patient comfortable, expecting, if necessary, to see her again. In the course of four weeks I was sent for, and found her, if possible, in a more suffering condition than ever, the spasms and pains being as severe as in many cases of parturition, but without the relief that attends them. No discharge per vaginam appeared during the whole time.

My mind was led to insist on the necessity of examining the vagina, which was permitted, and I was surprised to find it entirely impervious, constituting a complete atresia, or obstruction of a natural passage. I discovered, upon extending the examination, that the vagina was closed by a strong membrane, no doubt the result of an inflammation. There was a moderate swelling of the hypogastrium, such as would appear at the fourth or fifth month of pregnancy. There was no obstruction in the discharge of the urine.

Reasoning on the case, I came to the conclusion that the disappearance of the menstrual discharge was occasioned by the obliteration of the vaginal passage; and that, most likely, all the sufferings and distress of the patient were owing to an accumulation of the menstrual fluid in several successive menstruations; and that, at every catamenial period, an effort of nature to expel the offending fluid took place. Upon ocular examination, I could distinctly perceive the membrane of a dark blue tint, which determined me to pronounce that an accumulation of some fluid existed, and that it could be relieved by dividing the membrane. Agreeably to my invitation, I met, on the next morning, my friend Dr. Darlington, of West Chester, who united with me as to the necessity for an operation.

The patient was placed upon her back, her knees drawn upwards and separated; Dr. D. separated the parts, so as to enable me to see the membrane clearly, and select the point for the incision. I divided the membrane with a common scalpel, and to our mutual astonishment, a strong jet of grumous blood, which flew two or three feet from the orifice, followed the division. It was of the consistence of tar or molasses, and continued to flow until five or six pounds or even more was discharged, and continued to escape less rapidly during several hours.

The operation was followed by immediate relief; and, in due time, by a perfect restoration of the health; none of the unpleasant symptoms ever again appearing. She afterwards menstruated healthily. Care was taken to prevent the reunion of the membrane.

In the course of eight or ten months she became pregnant; I attended her during the labour, but nothing presented during the period worthy of notice. She has since been married to the father of the child, has had several children, and is doing well.

